# Self-Reported Disordered Eating and Weight-Control Behaviours Across BMI Categories in Romanian Young Adults: A Cross-Sectional Online Convenience Survey

**DOI:** 10.3390/nu18162677

**Published:** 2026-08-16

**Authors:** Alexandru Mischie, Viorel Jinga, Aniela-Roxana Nodiți-Cuc, Ramona Amina Popovici, Diana Marian, Gabriela Elena Strete, Andreea Mihaela Kis, Alexandra Enache, Laria-Maria Trusculescu, Anca Evelina Bolborea, Calin Muntean, Norina Consuela Forna, Liana Dehelean

**Affiliations:** 1 Doctoral School, “Victor Babeș” University of Medicine and Pharmacy, 300041 Timișoara, Romania; alexandru.mischie@umft.ro; 2 Faculty of Medicine, “Carol Davila” University of Medicine and Pharmacy, 020021 Bucharest, Romania; viorel.jinga@umfcd.ro; 3 Department of Surgical Oncology, “Carol Davila” University of Medicine and Pharmacy, 020021 Bucharest, Romania; 4 Department of Management and Communication in Dental Medicine (Department I), Faculty of Dental Medicine, “Victor Babeș” University of Medicine and Pharmacy of Timișoara, 300041 Timișoara, Romania; kis.andreea@umft.ro (A.M.K.); laria.trusculescu@umft.ro (L.-M.T.); 5 Department of Dentistry, Faculty of Dentistry, “Vasile Goldiș” Western University of Arad, 94–96 Revoluției Blvd., 310025 Arad, Romania; marian.diana@uvvg.ro; 6 Department of Psychiatry, “George Emil Palade” University of Medicine, Pharmacy, Science and Technology of Târgu Mureș, 540142 Târgu Mureș, Romania; elena.buicu@umfst.ro; 7 Discipline of Legal Medicine, Bioethics, Deontology and Medical Law, “Victor Babeș” University of Medicine and Pharmacy of Timișoara, Eftimie Murgu Sq., 300041 Timișoara, Romania; enache.alexandra@umft.ro; 8 Timiş County Emergency Clinical Hospital “Pius Brînzeu”, Liviu Rebreanu Bvld. 156, 300723 Timișoara, Romania; anca.bolborea@gmail.com; 9 Department III—Functional Sciences, Medical Informatics and Biostatistics, “Victor Babeș” University of Medicine and Pharmacy, 300041 Timișoara, Romania; cmuntean@umft.ro; 10 Department of Implantology, Removable Prostheses and Dental Prostheses Technology, Faculty of Dental Medicine, “Grigore T. Popa” University of Medicine and Pharmacy, 700115 Iași, Romania; norina.forna@umfiasi.ro; 11 Neuroscience Department, Faculty of Medicine, “Victor Babeș” University of Medicine and Pharmacy, 300041 Timișoara, Romania; lianadeh@umft.ro

**Keywords:** disordered eating, emotional eating, night eating, weight-control behaviours, body image, body mass index, nutritional status, young adults, Romania, public health nutrition

## Abstract

Background/Objectives: Disordered eating behaviours are increasingly common among young adults, yet nutrition-focused, region-specific data from Romania and Central and Eastern Europe (CEE) remain scarce. We characterised nutritional patterns, weight-control practices, disordered-eating indicators, body image, and psychosocial factors among Romanian young adults and tested how these differed across body mass index (BMI) categories. Methods: In a cross-sectional online survey (March–April 2026), 753 Romanian adults aged 18–30 years (93.5% female) completed a 30-item questionnaire. Self-reported anthropometric measurements were classified using WHO BMI cutoffs. Prevalences (with Wilson 95% confidence intervals [CIs]) were compared across BMI categories using the chi-square test, and multivariable logistic regression, adjusted for age and sex, estimated odds ratios (ORs). Results: Most participants were normal weight (59.1%); 11.6% were underweight, 29.3% were overweight or obese, and 86.3% were omnivorous. Dietary restriction (57.4%) and physical exercise (46.7%) were the leading weight-control methods; self-induced vomiting (6.4%) and medication (4.5%) were less frequent. Lifetime compulsive/emotional eating was reported by 50.1%, current stress-related eating by 49.1%, and guilt after eating by 66.1%. Lifetime night eating reached 31.9% (current, 12.9%). Body dissatisfaction (36.1% “not at all satisfied”), difficulty looking in the mirror (66.7%) and self-reported stress (82.2%) were widespread. In adjusted models, dietary restriction, emotional eating and guilt after eating rose steeply and monotonically with BMI (overweight/obesity vs. normal weight: aOR 2.34, 2.49 and 2.73, respectively; all *p* < 0.001), whereas current night eating and frequent self-weighing did not differ across BMI categories (*p* = 0.92 and *p* = 0.82). Dietary restriction increased from 24.1% in underweight to 55.5% in normal-weight and 74.2% in overweight/obesity participants; corresponding gradients were also observed for emotional eating and food-related guilt. Conclusions: In this predominantly female, largely urban online convenience sample of Romanian young adults, self-reported behavioural risk indicators related to disordered eating were common. Restriction, emotional eating and food-related guilt increased with BMI, whereas night eating and self-weighing showed no clear BMI gradient. These findings should be interpreted cautiously and should not be generalised to Romanian young men or rural populations without confirmation in more representative samples.

## 1. Introduction

Feeding and eating disorders (EDs) and the broader spectrum of subthreshold disordered eating behaviours constitute a growing public health and clinical nutrition concern worldwide. A systematic review covering 2000–2018 reported that the weighted mean point prevalence of EDs rose from 3.5% in 2000–2006 to 7.8% in 2013–2018, with consistently higher rates in women [1]. Subsequent meta-analytic work confirms that EDs are most common in Western settings and in female populations, and that newer categories—including binge-eating disorder (BED)—are substantially more prevalent than the historically dominant anorexia nervosa and bulimia nervosa [2]. Across Europe, narrative syntheses estimate that up to 4% of women report anorexia nervosa, up to 2% bulimia nervosa, up to 4% BED, and a further 2–3% subthreshold presentations, with most cases remaining undetected by health services [3]. Nationally representative data from the United States, using DSM-5 criteria [4], likewise demonstrate clinically meaningful lifetime prevalences and their association with obesity and psychosocial impairment [5].

The burden of disordered eating extends well beyond the relatively small proportion of individuals who meet full diagnostic criteria. Sub-clinical behaviours—chronic dietary restriction, emotional or stress-related eating, night eating, compensatory practices and compulsive self-weighing—are far more prevalent than diagnosable EDs and are themselves nutritionally consequential. These behaviours reshape meal timing, food selection, energy distribution across the day and dietary adequacy, and they frequently coexist with body dissatisfaction and psychological distress [6,7,8]. From a nutritional standpoint, this matters across the entire weight spectrum: restrictive patterns can compromise micronutrient adequacy and lean mass, whereas emotional and night eating are associated with disordered energy regulation and a higher likelihood of overweight and obesity. A recent meta-analysis of 21,237 people found a pooled prevalence of emotional eating of 44.9% in overweight and obese populations [9]; a systematic review confirmed that emotional eating is significantly greater among adults living with obesity than among those with a healthy BMI [8], and stress shifts intake toward energy-dense, nutrient-poor foods and away from healthier options [7]. Night eating syndrome, defined by an abnormal shift in intake to the evening and night, further links circadian-misaligned eating to metabolic and psychological morbidity [6].

The nutritional dimension of disordered eating is frequently overshadowed by its psychiatric framing, and most epidemiological evidence originates from Western Europe, North America, Asia and Oceania. Data from Romania and the wider CEE region remain notably scarce. This gap is consequential in a country where overweight and obesity among the young are rising, dedicated dietetic and eating-disorder services are limited and unevenly distributed, and the mental-health burden increased after the COVID-19 pandemic; a recent meta-analysis estimated a global point prevalence of eating disorders of 5.23% among children and young people, with presentations rising during and after the pandemic [10]. A recent nationwide survey of 1202 Romanian young adults found a 32.4% prevalence of overweight/obesity and identified compulsive eating as one of the most consistent behavioural correlates of adiposity [11], underscoring the need for nutrition-focused characterisation of eating behaviours in this population. Comparable evidence from Central–Eastern Europe is limited, although a recent Polish study linked emotionally driven eating and body-image concerns to disordered-eating behaviours in adolescents [12]. Studies that explicitly describe nutritional behaviours and weight-control practices—rather than diagnostic categories alone—are rarer still. Dietitians and nutritionists are often the first professionals to encounter restrictive dieting, emotional eating or night eating in routine practice. Yet, they lack region-specific data to inform screening, risk stratification and counselling. Characterising how nutritionally relevant behaviours differ across BMI categories is particularly valuable, because it can reveal distinct behavioural patterns that may be relevant to screening and counselling and that a single overall prevalence figure may obscure.

Accordingly, this study had three aims: (i) to describe the prevalence of nutritional behaviours, weight-control methods, disordered-eating indicators, body image and psychosocial factors in a relatively large sample of Romanian young adults; (ii) to test how these behaviours differ across BMI categories (underweight, normal weight, overweight/obesity), with particular attention to contrasting risk profiles at the low and high ends of the distribution; and (iii) to translate the findings into practical implications for nutrition professionals and public health nutrition in Romania and the CEE region.

We hypothesised that nutritionally relevant behavioural risk indicators would differ across BMI categories, with higher frequencies of dietary restriction, emotional eating, food-related guilt, and body-image concerns among participants with overweight/obesity compared with those of normal weight. We further hypothesised that some behaviours, such as night eating and frequent self-weighing, might show weaker or no clear BMI-related gradients.

## 2. Materials and Methods

### 2.1. Study Design and Setting

This was a cross-sectional, questionnaire-based study conducted online in Romania over two months (March–April 2026). The survey was disseminated electronically through social media platforms and university and community networks, and responses were collected anonymously via a self-administered web form.

Reporting followed the STROBE statement for cross-sectional studies and the CHERRIES checklist for internet e-surveys. The survey was open-access and did not require registration or login. To reduce duplicate submissions, the online platform was configured to allow one response per browser session. Because the number of individuals who accessed the survey link without completing the questionnaire was not recorded, a participation or completion rate could not be calculated. As participation was voluntary and recruitment relied on social media and university/community networks, voluntary-response and self-selection bias cannot be excluded.

### 2.2. Participants

Eligible participants were young adults aged 18–30 years residing in Romania who provided informed consent and completed the questionnaire. Recruitment used a non-probability convenience strategy; participation was voluntary and uncompensated. A total of 753 valid responses were analysed. The sample was predominantly female *(n* = 704, 93.5%; male, n = 49, 6.5%) and predominantly urban (urban, *n* = 509, 67.6%; rural, *n* = 244, 32.4%), with a mean age of 22.5 ± 3.4 years. The marked female predominance is consistent with the well-documented over-representation of women in online surveys on eating behaviour and body image and is addressed in the limitations.

### 2.3. Questionnaire and Variable Definitions

Data were collected with a 30-item self-report questionnaire developed for this study and administered in Romanian, structured into five domains: (1) demographics (age, sex, residence, education, and self-reported height and weight); (2) nutritional behaviours and meal patterns (diet type; number of main meals and snacks; weekly physical-activity level; night eating); (3) weight-control methods; (4) body image; and (5) psychosocial factors. Response formats and the coding used for the key variables are specified below.

Item generation was guided by the study objectives and by commonly reported eating-, weight-control-, body-image-, and stress-related behaviours in young adults. Before dissemination, the instrument underwent preliminary face and content review by a multidisciplinary panel of 5 experts in nutrition, public health, psychology/psychiatry, biostatistics, and medical research. Experts assessed the relevance, clarity, and comprehensibility of each item, and minor wording revisions were made accordingly. The questionnaire was subsequently pilot-tested in 20 young adults to evaluate item clarity, completion time, and technical functionality. Pilot responses were not included in the final analysis. The instrument was not designed to establish clinical diagnoses and was not subjected to full psychometric validation against established multi-item instruments. The complete questionnaire is provided in the Supplementary Materials (Questionnaire S1).

Weight-control methods (“Which methods have you used to lose weight or avoid gaining weight?”) were captured using a multiple-response item that offered self-induced vomiting, medication, dietary restriction, physical exercise, or “I have not used such methods”; each option was coded as endorsed vs. not endorsed. Night eating was assessed both as a lifetime experience (listed among prior disordered-eating experiences) and as a current behaviour (“Do you usually wake up at night to eat?”; current = “sometimes” or “often” vs. “no”). Self-weighing frequency (“How often do you usually weigh yourself?”) offered five ordered options—“before and after every meal”, “once a day”, “weekly”, “rarely” and “I do not weigh myself”—and was dichotomised as frequent self-weighing (at least weekly, i.e., weekly, daily, or before/after every meal) vs. infrequent (rarely or never). Diet type was distinguished between omnivorous, vegetarian, ovo-lacto-vegetarian, meat-based, and other patterns. Body dissatisfaction was defined as responding “not at all satisfied” to the question “How satisfied are you with the way you look?” Perceived psychological problems (including stress) were assessed using a multiple-response item, and self-reported stress was defined as endorsement of “stress”.

Nine statements on eating, weight and body image (items 21–29) were rated on a 0–4 frequency scale (0 = never, 1 = rarely, 2 = sometimes, 3 = often, 4 = always). For analysis, each item was dichotomised as present (score ≥ 1, i.e., experienced at least rarely) vs. absent (score 0, never). This yielded the indicators of guilt after eating (item 25), loss of control over eating (item 26), fear of weight gain (item 21), eating avoidance despite hunger (item 22), difficulty looking in the mirror (item 27) and negative influence of social media on body image (item 29). A final agree/apply checklist (item 30) captured emotional (stress-related) eating (“When I feel sad or stressed I eat without realising it”), the impact of negative appearance-related comments on self-confidence, and a rigid belief in dietary control. The instrument was designed for accessibility to a general (non-clinical) audience; it therefore uses single, study-specific items rather than validated multi-item instruments, a design choice discussed in the limitations.

### 2.4. Anthropometry and BMI Categorisation

BMI was computed from self-reported height and weight (kg/m^2^) and classified using WHO cut-offs: underweight (<18.5), normal weight (18.5–24.9), overweight (25.0–29.9) and obesity (≥30.0). In this sample, 87 participants (11.6%) were underweight, 445 (59.1%) were normal weight, 137 (18.2%) were overweight, and 84 (11.2%) were obese. For stratified analyses, overweight and obesity were combined into a single “overweight/obesity” category (n = 221, 29.3%) to preserve cell sizes and to contrast the low, middle and high ends of the distribution. All records had complete, biologically plausible anthropometric values (weight 40–125 kg; height 148–200 cm), so none were excluded.

### 2.5. Statistical Analysis

Categorical variables were summarised as counts and percentages, and continuous variables as means ± standard deviations. Wilson 95% CIs were computed for key prevalences. Because several items permitted multiple responses, the corresponding category percentages may sum to more than 100%. Associations between BMI category and each key behaviour were tested with the Pearson chi-square test; Fisher’s exact test was reserved for tables with expected counts <5 (not required here, as all expected counts exceeded 5). For each behaviour, a multivariable binary logistic regression was fitted with BMI category (normal weight as reference) as the exposure and age (years) and sex as covariates, yielding adjusted odds ratios (aORs) with 95% CIs. A two-sided *p* < 0.05 was considered statistically significant. Analyses were performed in Python 3.11 using pandas 3.0.4, statsmodels 0.14 and SciPy 1.17. 

As a sensitivity analysis, five key frequency-based behavioural indicators were re-dichotomised using a stricter threshold of ≥2 (“sometimes” or more frequently), with scores of 0–1 classified as absent and scores of 2–4 as present. BMI-group comparisons were repeated using chi-square tests, and age- and sex-adjusted logistic regression models were refitted using the stricter threshold.

As an additional exploratory contextual analysis, the five principal behavioural outcomes were compared between urban and rural participants using Pearson chi-square tests. Cramér’s V was calculated as a measure of effect size. Because five residence-related comparisons were performed, Holm-adjusted *p*-values were also considered when interpreting these exploratory results.

No formal correction for multiple comparisons was applied, and the analyses should be regarded as exploratory.

### 2.6. Ethics

The study was conducted in accordance with the Declaration of Helsinki. The survey was anonymous and voluntary, collected no directly identifying data, and all participants provided informed consent electronically before accessing the questionnaire. It was reviewed by the Committee for Scientific Research Ethics of the “Victor Babeș” University of Medicine and Pharmacy Timișoara (approval no. 59/02.03.2026). Data collection commenced on 10 March 2026, after ethics approval had been obtained, and continued through April 2026.

## 3. Results

### 3.1. Demographics and BMI Distribution

A total of 753 young adults aged 18–30 years were included (mean age 22.5 ± 3.4 years; 93.5% female; 67.6% urban). Mean BMI was 23.4 ± 4.8 kg/m^2^. Based on self-reported anthropometric data, 59.1% were normal weight, 11.6% were underweight, and 29.3% were overweight or obese (18.2% overweight, 11.2% obese). The great majority followed an omnivorous diet (86.3%), with the remainder reporting meat-based, ovo-lacto-vegetarian, vegetarian or other patterns. Two main meals per day were most common (55.1%), and 29.7% reported no weekly physical activity. The sample composition differed from that expected in the general Romanian young-adult population, particularly because of the marked predominance of women (93.5%) and the high representation of urban residents (67.6%). These differences should be considered when interpreting the external validity of the findings.

### 3.2. Nutritional Patterns and Weight-Control Methods

**Figure 1:**
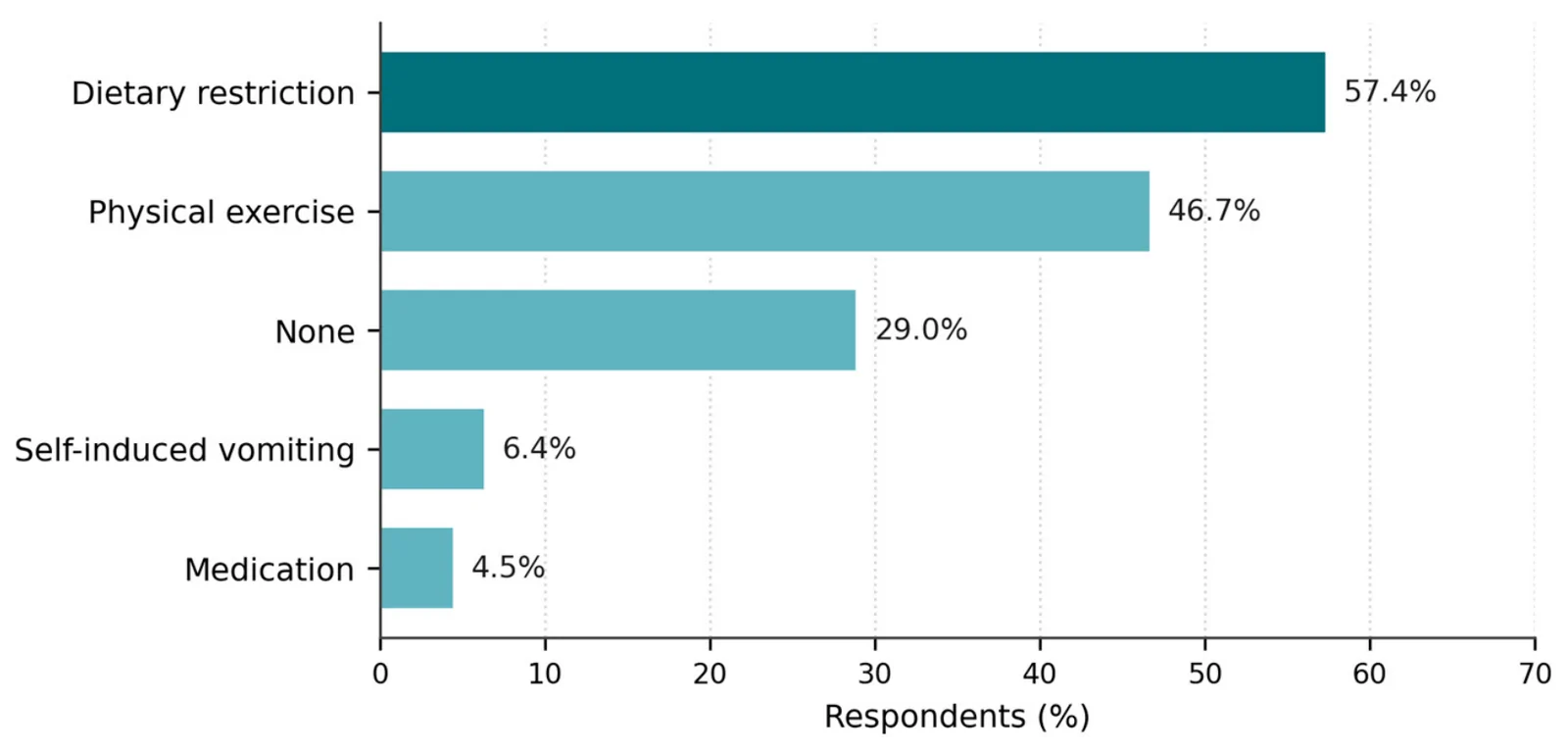
Prevalence of weight-control methods among Romanian young adults (N = 753). Percentages exceed 100% because participants could select more than one method. “None” indicates respondents who did not endorse any of the listed weight-control methods.

Weight-control practices were common and frequently combined (multiple-response item). Dietary restriction was the most prevalent method (57.4% (95% CI 53.8–60.9%)), followed by physical exercise (46.7% (95% CI 43.2–50.3%)). Almost three in ten respondents (29.0% (95% CI 25.9–32.3%)) reported using no specific weight-control method. Compensatory or pharmacological practices were less common but clinically important: self-induced vomiting was reported by 6.4% (95% CI 4.9–8.4%) and medication by 4.5% (95% CI 3.2–6.2%) (Figure 1). Altered meal timing was also evident: night eating had been experienced by 31.9% (95% CI 28.7–35.3%) of participants at some point, and 12.9% (95% CI 10.7–15.5%) reported it as a current behaviour.

### 3.3. Disordered-Eating Indicators and Body Image

Self-reported behavioural risk indicators related to disordered eating were common in the sample. Half of the sample (50.1% (95% CI 46.5–53.7%) reported lifetime compulsive/emotional eating, 49.1% (95% CI 45.5–52.7%) reported currently eating in response to sadness or stress, and 74.4% (95% CI 71.2–77.4%) reported loss of control over eating. Notably, 66.1% (95% CI 62.7–69.4%) reported guilt after eating and 88.0% (95% CI 85.5–90.2%) reported fear of gaining weight, pointing to a pervasive negative affective relationship with food. Body-image disturbance was equally striking: 36.1% (95% CI 32.7–39.6%) were “not at all satisfied” with their appearance, 66.7% (95% CI 63.3–69.9%) found it difficult to look at themselves in the mirror, and 77.6% (95% CI 74.4–80.4%) felt that social media negatively influenced their body image.

### 3.4. Psychosocial Factors

Perceived psychological burden was high: 82.2% (95% CI 79.3–84.8%) of participants reported stress, and 46.1% (95% CI 42.6–49.7%) indicated that negative comments about their appearance had affected their self-confidence; The observed proportions were 46.2% among women and 44.9% among men (*p* = 0.98); however, the small male subsample (n = 49) precludes meaningful conclusions regarding sex differences. A rigid dietary-control belief (“I cannot reach my ideal shape unless I have complete control over my eating”) was endorsed by 48.9% (95% CI 45.3–52.5%) of participants. The co-occurrence of self-reported stress, body dissatisfaction and emotional/compulsive eating is consistent with stress-driven dysregulation of eating behaviour. Descriptive prevalences and Wilson 95% CIs for all nine frequency-based behavioural risk indicators are provided in Supplementary Table S2.

### 3.5. Associations Between BMI Categories and Key Behaviours

**Figure 2:**
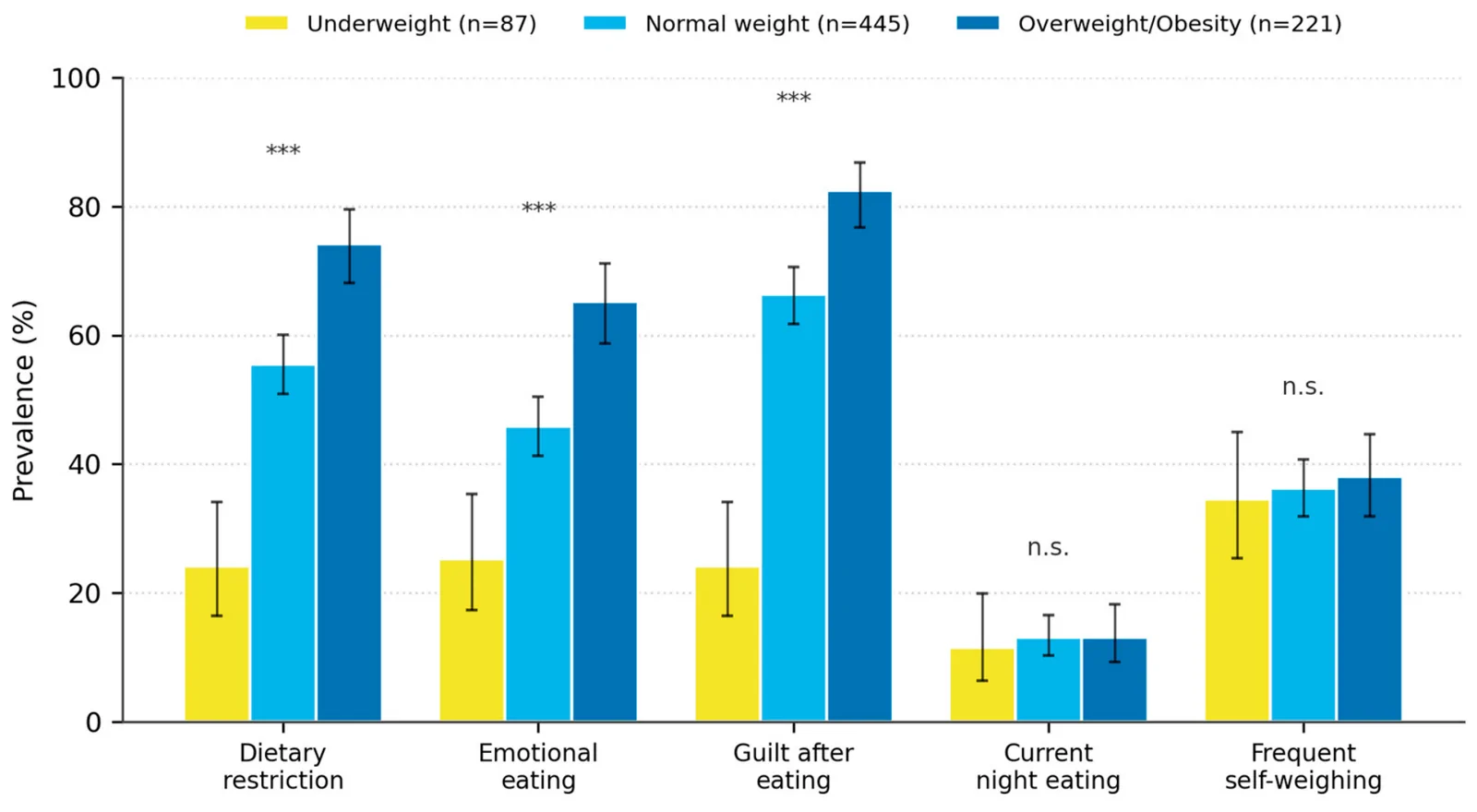
Observed prevalence of key nutritional and disordered-eating behaviours by BMI category. Bars show within-category prevalence; error bars are Wilson 95% confidence intervals. Dietary restriction, emotional eating and guilt after eating increase significantly with BMI, whereas current night eating and frequent self-weighing do not differ across categories. Significance of the chi-square test across the three groups: *** *p* < 0.001; n.s., not significant. The colour palette is colour-blind accessible.

The age- and sex-adjusted associations, including aORs, 95% CIs, and *p*-values, are reported in Table 1, whereas the corresponding observed prevalences across BMI categories with Wilson 95% CIs are presented in Figure 2. A clear and consistent pattern emerged: dietary restriction, emotional eating and guilt after eating all increased steeply and monotonically with BMI. Dietary restriction rose from 24.1% in underweight to 55.5% in normal-weight and 74.2% in overweight/obese participants (*p* < 0.001); emotional eating from 25.3% to 65.2% (*p* < 0.001); and guilt after eating from 24.1% to 82.4% (*p* < 0.001). After adjustment for age and sex, overweight/obese participants had markedly higher odds of restriction (aOR 2.34 (1.63–3.36)), emotional eating (aOR 2.49 (1.76–3.52)) and guilt (aOR 2.73 (1.80–4.13)) than normal-weight participants, while underweight participants had significantly lower odds of all three (e.g., guilt aOR 0.15 (0.09–0.26)). In contrast, current night eating and frequent self-weighing were common but did not differ across BMI categories (night eating 11.5–13.1%, *p* = 0.92; self-weighing 34.5–38.0%, *p* = 0.82). Thus, rather than the two opposing gradients sometimes anticipated from the eating-disorder literature, the data show that affect- and control-related eating behaviours concentrate at the higher end of the BMI distribution. In contrast, meal timing and self-monitoring behaviours showed no clear BMI-related gradient in this young adult sample.

Sensitivity analyses using the stricter threshold of ≥2 (“sometimes” or more frequently) yielded lower absolute prevalences, as expected, while preserving the overall BMI-related gradients. Fear of weight gain, eating avoidance despite hunger, guilt after eating, loss of control over eating, and difficulty looking in the mirror all remained significantly associated with BMI category (all *p* < 0.001). In age- and sex-adjusted analyses, participants with overweight/obesity had higher odds of all five behavioural risk indicators compared with normal-weight participants (Supplementary Table S1).

In exploratory analyses stratified by residence, dietary restriction, emotional eating, and guilt after eating, these variables showed similar prevalences among urban and rural participants. Current night eating was more frequently reported by rural than urban participants (17.2% vs. 10.8%; *p* = 0.014; Cramér’s V = 0.090), whereas frequent self-weighing was somewhat less frequent in rural participants (31.6% vs. 38.9%; *p* = 0.050). However, these effects were small, and the residence-related association with night eating did not remain statistically significant after adjustment for the five exploratory comparisons. Full results are presented in Supplementary Table S3.

## 4. Discussion

### 4.1. Principal Findings

In this cross-sectional survey of 753 Romanian young adults, nutritionally relevant disordered-eating behaviours were strikingly common. Half of the sample reported lifetime compulsive/emotional eating, half currently ate in response to negative emotions, and two-thirds experienced guilt after eating; dietary restriction was the dominant weight-control strategy, and body dissatisfaction and self-reported stress were near-ubiquitous. The BMI-stratified analysis revealed a coherent gradient rather than two opposing ones: dietary restriction, emotional eating and food-related guilt all increased steeply and monotonically with BMI and remained strongly associated after adjustment for age and sex, whereas current night eating and frequent self-weighing were common but showed no clear BMI-related gradient. This suggests that disordered eating in young adults may encompass distinct behavioural patterns, with affect- and weight-control-related behaviours concentrated at the higher end of the BMI spectrum.

### 4.2. Nutritional Implications Across the BMI Spectrum

The observed gradients carry direct nutritional meaning. The concentration of dietary restriction, emotional eating and guilt in overweight and obese participants is consistent with a self-reinforcing cycle in which weight concern drives restrictive attempts, restriction and negative affect precipitate loss-of-control or emotional eating, and guilt maintains the cycle—patterns that tend to displace intake toward energy-dense, nutrient-poor foods and undermine diet quality and weight management. These patterns are compatible with previously proposed mechanisms linking stress, restrictive eating, emotional eating, and obesity; however, the cross-sectional design of the present study does not permit conclusions regarding temporal sequence or causality. This aligns with meta-analytic evidence that emotional eating is substantially more prevalent among people with overweight and obesity [8,9] and with national data linking compulsive eating to adiposity in Romanian young adults [11]. It also converges with syntheses of obesity treatment-seeking samples, in which binge-type and other disordered eating substantially exceed community norms and high body weight and eating pathology share common aetiological drivers [13]. Importantly, restriction was least common—not most common—among underweight participants, indicating that in a general (non-clinical) young-adult population, restrictive weight-control behaviour is driven mainly by higher body weight and weight concern rather than by low weight per se; the restriction–underweight link characteristic of clinical anorexia nervosa is not the dominant signal at the population level. Nonetheless, the substantial minority of underweight participants who restrict, self-weigh frequently or eat at night still merit clinical attention, because restriction at low body weight carries the greatest risk of energy and micronutrient inadequacy and loss of lean mass. The near-uniform high perceived stress (82.2%) suggests a plausible common pathway: stress reliably shifts eating toward unhealthy options [7] and may act as an upstream driver that manifests differently across individuals. However, stress was assessed using a single self-reported item, and the high observed prevalence may also reflect voluntary-response and self-selection bias; therefore, it should not be interpreted as the population prevalence of clinically significant stress. By contrast, the absence of a BMI gradient for current night eating in our sample differs from previous clinical and epidemiological evidence linking night-eating syndrome and related nocturnal eating patterns to obesity [14]. Several factors may explain this discrepancy. Our measure was based on a single self-reported item assessing current nocturnal eating rather than a validated instrument designed to identify night-eating syndrome, and it did not capture key diagnostic dimensions such as evening hyperphagia, frequency, duration, associated distress, or awareness of nocturnal eating episodes. In addition, the relatively young, non-clinical and self-selected nature of our sample may differ substantially from populations in which stronger associations with obesity have been observed. Therefore, the lack of a BMI gradient in the present study should not be interpreted as evidence that night-eating behaviours are unrelated to adiposity.

### 4.3. Comparison with International and Regional Data

The behavioural prevalences observed here are high relative to the diagnosable ED prevalences reported internationally—point prevalences of roughly 2–6% in women [1], European ranges generally below 4% per disorder [3], and lifetime DSM-5 estimates of ~1–2% in representative samples [2,5]. This contrast is expected: our instrument captured subclinical, nutritionally relevant behaviours rather than diagnostic categories, and such behaviours are far more common than full-syndrome EDs, yet still degrade diet quality and well-being. Our current prevalence of stress-related emotional eating (49.1%) is broadly consistent with the pooled 44.9% reported for overweight/obese populations [9], and the positive emotional-eating–BMI gradient aligns closely with dedicated meta-analytic findings [8]. The overall prevalence of overweight/obesity (29.3%) is comparable to the 32.4% recently reported in a nationwide sample of Romanian young adults [11]. Given the scarcity of Romanian/CEE evidence, these data provide an early, nutrition-focused benchmark for the region. The present findings are also consistent with recent Romanian data from university students, in which eating habits, body weight perception and psycho-emotional factors were closely interrelated. This supports the relevance of integrating nutritional, psychological and body-image dimensions when assessing young adults in Romania [15].

### 4.4. Implications for Nutrition Professionals and Public Health

Several practice implications follow. First, the findings suggest that non-stigmatising screening for these behaviours may be useful in dietetic and primary-care settings, although its clinical utility requires further evaluation. Second, the gradients argue for BMI-tailored counselling: weight-restorative, flexible-eating and micronutrient-adequacy strategies for the minority restricting at low weight; and, at higher BMI, structured support for emotion regulation, meal structure and food-related guilt as part of weight management, alongside broader dietary and lifestyle strategies for obesity [16,17,18]. Third, the high burden of guilt and body dissatisfaction supports weight-inclusive, non-diet approaches that target the relationship with food, such as fostering intuitive eating competencies, which are associated with reduced disordered eating and improved well-being [19]. Given the increasing use of digital health tools, future studies could evaluate the feasibility of incorporating brief validated screening tools into digital health settings, including university health services, primary care, and dietetic practice [20,21]. Such approaches would require prospective evaluation of feasibility, acceptability, equity, and clinical utility before broader implementation could be recommended [22,23]. Low-burden digital self-help and clinician-supported online interventions have shown promise for mild-to-moderate disordered eating and may warrant further evaluation in settings with limited specialist services [24,25]. Finally, the combination of self-reported stress, appearance-related pressures and disordered eating points to a need for campus- and community-based interventions integrating nutrition literacy, stress management and body-image education—particularly given the well-documented contribution of social media to body dissatisfaction and disordered eating in young people [26,27,28], echoed by the 77.6% of our sample who felt negatively influenced by social media—and for incorporating these competencies into dietitian and health-professional training in Romania and the CEE region.

### 4.5. Strengths and Limitations

Key strengths of this study include the relatively large sample size (N = 753), the explicit focus on eating- and weight-control-related behavioural indicators, the BMI-stratified analytical approach, the use of age- and sex-adjusted regression models, and the provision of data from a relatively understudied Romanian and Central and Eastern European context. The questionnaire also underwent multidisciplinary expert review and pilot testing before dissemination.

Several limitations should, however, be considered when interpreting the findings. First, recruitment relied on an online convenience sampling strategy through social media, university, and community networks. This approach may have introduced substantial volunteer and self-selection bias, because individuals with greater interest in eating, weight, body image, stress, or related health concerns may have been more likely to participate. Such self-selection may also have contributed to the high observed prevalences of body dissatisfaction, perceived stress, and other behavioural risk indicators. Accordingly, these estimates should not be interpreted as representative population prevalences. The marked female predominance (93.5%) and urban majority (67.6%) further limit generalisability, particularly to Romanian young men and rural residents. Although sex was included as a covariate in the regression models, the small male subsample (n = 49) limits the precision of sex adjustment and precludes robust sex-specific inference; residual confounding related to the marked sex imbalance cannot be excluded.

Second, height and weight were self-reported and were therefore susceptible to reporting error. Self-reported weight may be underestimated, and height overestimated, potentially resulting in BMI misclassification [29,30]. Such errors may have shifted some participants across BMI-category boundaries and could have attenuated or distorted the observed BMI–behaviour associations.

Third, although the study-specific questionnaire underwent expert review and pilot testing, it was not subjected to formal psychometric validation against established instruments. Several constructs were assessed using single items rather than validated multi-item measures such as the EDE-Q or the Night Eating Questionnaire. This limits measurement precision, construct validity, and comparability with studies using validated instruments and does not fully capture the multidimensional nature or severity of the underlying constructs. The reported prevalences should therefore be interpreted as self-reported behavioural risk indicators rather than clinical caseness. In addition, the 0–4 frequency items were primarily dichotomised at a lenient threshold of ≥1 (“at least rarely”), which captures any occurrence rather than clinically meaningful frequency; this limitation was addressed through a sensitivity analysis using a stricter threshold of ≥2 (“sometimes” or more frequently).

Fourth, the study did not include quantitative assessment of dietary intake, total energy intake, macronutrient or micronutrient consumption, or overall diet quality. Consequently, the nutritional implications of the observed behaviours are inferred from self-reported behavioural indicators rather than directly demonstrated. The findings should therefore not be interpreted as evidence of actual dietary inadequacy, nutrient deficiency, or altered energy intake.

Fifth, no a priori sample-size calculation was performed, and multiple statistical comparisons were conducted without formal adjustment for multiplicity. The analyses should therefore be regarded as exploratory, and the possibility of type I error cannot be excluded, although the principal associations were supported by very small *p*-values and the main null findings were clearly non-significant.

Finally, the cross-sectional design precludes assessment of temporal sequence and causal relationships between BMI, psychosocial factors, and eating-related behaviours. Mechanistic interpretations regarding stress, emotional eating, dietary restriction, body-image concerns, and obesity should therefore be regarded as hypothesis-generating rather than causal. Longitudinal studies using more representative samples, validated psychometric instruments, measured anthropometry, and direct dietary assessment are required to confirm and extend these findings.

### 4.6. Future Directions

Future work should (a) employ validated instruments (EDE-Q, DEBQ, Night Eating Questionnaire) to benchmark against clinical thresholds; (b) oversample men and rural residents and use probability-based or stratified recruitment; (c) incorporate measured anthropometry and quantitative dietary assessment to move from behavioural indicators to nutritional adequacy and diet quality; (d) pursue longitudinal designs linking stress, body image and meal-timing patterns to changes in BMI and dietary adequacy over time; and (e) pilot digital screening tools in appropriate healthcare and university-health settings to evaluate feasibility, equity, acceptability, and impact on early detection and referral.

## 5. Conclusions

Participants in this predominantly female and largely urban online convenience sample reported a high burden of self-reported behavioural risk indicators related to disordered eating, including emotional and compulsive eating, food-related guilt, dietary restriction, and night eating, alongside widespread body dissatisfaction and perceived stress. BMI-stratified analyses showed that dietary restriction, emotional eating, and guilt after eating increased with BMI and remained associated after adjustment for age and sex, whereas night eating and frequent self-weighing showed no clear BMI-related gradient. Given the non-probability sampling strategy and the observational, cross-sectional design, these findings should be interpreted with caution and not considered representative of the general Romanian young-adult population. They may nevertheless help identify areas for further investigation and inform the development of future screening and prevention strategies, subject to confirmation in more representative populations. Future studies should prioritise probability-based or stratified sampling, a more balanced sex distribution, validated multi-item eating-behaviour instruments, measured anthropometry, direct dietary assessment, and longitudinal designs to better establish temporal relationships and improve generalisability.

## Figures and Tables

**Table 1:** Age- and sex-adjusted associations between BMI category and key nutritional and disordered-eating behavioural indicators. Adjusted odds ratios (aORs), 95% confidence intervals (CIs), and exact *p*-values are presented. Normal weight was used as the reference BMI category. Observed prevalences across BMI categories with Wilson 95% CIs are shown separately in Figure 2.

Behaviour	Underweight vs. Normal aOR (95% CI)	*p*	Overweight/Obesity vs. Normal aOR (95% CI)	*p*
Dietary restriction	0.25 (0.15–0.43)	<0.001	2.34 (1.63–3.36)	<0.001
Emotional eating	0.38 (0.23–0.64)	0.0003	2.49 (1.76–3.52)	<0.001
Guilt after eating	0.15 (0.09–0.26)	<0.001	2.73 (1.80–4.13)	<0.001
Current night eating	0.84 (0.41–1.71)	0.629	1.06 (0.66–1.73)	0.800
Frequent self-weighing	0.94 (0.58–1.52)	0.798	1.07 (0.76–1.50)	0.702

aOR, adjusted odds ratio; CI, confidence interval. Normal weight was used as the reference BMI category. Models were adjusted for age and sex. Observed prevalences across BMI categories with Wilson 95% confidence intervals are presented separately in Figure 2.

## Data Availability

The data presented in this study are available on reasonable request from the corresponding author due to privacy and ethical restrictions.

## Supplementary Materials

The following supporting information can be downloaded at: https://www.mdpi.com/article/10.3390/nu18162677/s1, Questionnaire S1: Eating Behaviours, Body Image, and Psychosocial Factors Questionnaire; Table S1: Sensitivity analysis of five behavioural risk indicators using a stricter frequency threshold of ≥2 (“sometimes” or more frequently), stratified by BMI category; Table S2: Prevalence of the nine frequency-based behavioural risk indicators using the primary threshold of ≥1 (“at least rarely”); Table S3: Exploratory comparison of key behavioural indicators according to urban versus rural residence.
