# Supplementary material for: Self-Reported Disordered Eating and Weight-Control Behaviours Across BMI Categories in Romanian Young Adults: A Cross-Sectional Online Convenience Survey
Source: Nutrients. 2026 Aug 16;18(16):2677. doi: 10.3390/nu18162677 (PMC13516123; doi:10.3390/nu18162677)
Supplement: Supplementary file 1 [file nutrients-18-02677-s001.zip › nutrients-4476104-supplementary.pdf]

Table S1. Sensitivity analysis of five behavioural risk indicators using a stricter frequency threshold of ≥2 ("sometimes" or more frequently), stratified by BMI category.

Behavioural risk indicator, ≥2	Underweight n=87	Normal weight n=445	Overweight/Obesity n=221	p	aOR Overweight/Obesity vs normal (95% CI)
Fear of weight gain	35.6%	78.0%	91.0%	<0.001	3.29 (1.93–5.60)
Eating avoidance despite hunger	17.2%	46.7%	59.3%	<0.001	1.76 (1.26–2.45)
Guilt after eating	14.9%	43.6%	71.0%	<0.001	3.62 (2.52–5.19)
Loss of control over eating	29.9%	50.1%	76.0%	<0.001	3.49 (2.41–5.07)
Difficulty looking in the mirror	24.1%	33.0%	60.2%	<0.001	3.29 (2.34–4.64)

Values are within-group prevalences. p-values are from Pearson chi-square tests across BMI categories. Adjusted odds ratios (aORs) were estimated using multivariable logistic regression with normal weight as the reference category and adjustment for age and sex.

Table S2. Prevalence of the nine frequency-based behavioural risk indicators using the primary threshold of ≥1 ("at least rarely").

Behavioural risk indicator	n/N	Prevalence, %	Wilson 95% CI
Fear of weight gain	663/753	88.0	85.5–90.2
Eating avoidance despite hunger	516/753	68.5	65.1–71.7
Told by others to eat more	411/753	54.6	51.0–58.1
Told by others to eat less	410/753	54.4	50.9–58.0
Guilt after eating	498/753	66.1	62.7–69.4
Loss of control over eating	560/753	74.4	71.1–77.4
Difficulty looking in the mirror	502/753	66.7	63.2–69.9
Comparing appearance with social-media images	617/753	81.9	79.0–84.5
Negative social-media influence on body image	584/753	77.6	74.4–80.4

Values represent self-reported behavioural risk indicators rather than clinical caseness. Items were rated on a 0–4 frequency scale and dichotomised using the primary threshold of ≥1 ("at least rarely"). CI, confidence interval.

Table S3. Exploratory comparison of key behavioural indicators according to urban versus rural residence.

Behaviour	Urban % (n)	Rural % (n)	p	Cramér's V
Dietary restriction	57.6 (293)	57.0 (139)	0.877	0.006
Emotional eating (sad/stressed)	50.3 (256)	46.7 (114)	0.359	0.033
Guilt after eating	66.8 (340)	64.8 (158)	0.579	0.020
Current night eating	10.8 (55)	17.2 (42)	0.014	0.090
Frequent self-weighing (≥ weekly)	38.9 (198)	31.6 (77)	0.050	0.071

Eating Behaviours, Body Image, and Psychosocial Factors Questionnaire

Please answer all questions. For items marked "Select all that apply," more than one response may be selected. Items 21–29 use the frequency scale shown below.

Section A. Demographic and Anthropometric Information
1. Please select your sex:
☐ Female
☐ Male
2. Please enter your age (completed years):
3. Area of residence:
☐ Urban
☐ Rural
4. Highest level of education completed:
☐ Lower secondary education
☐ Upper secondary education
☐ Post-secondary non-tertiary education
☐ Bachelor's degree
☐ Master's degree
☐ Doctoral degree
5. What is your body weight (kg)?
6. What is your height (cm)?
7. Do you have any chronic diseases? If yes, please specify the condition (e.g., diabetes mellitus or hypertension).
Section B. Eating-Related Knowledge and Dietary Patterns
8. Have you ever experienced an eating disorder or eating-related condition? If yes, please indicate which one(s): *(Select all that apply.)*
☐ No

☐ Anorexia nervosa
☐ Bulimia nervosa
☐ Compulsive/emotional eating
☐ Avoidant/restrictive food intake disorder (ARFID)
☐ Pica (ingestion of non-food substances)
☐ Rumination/regurgitation disorder
☐ Night eating
☐ Purging disorder
☐ Orthorexia
☐ Other:
9. Are you familiar with the concept of eating disorders (e.g., anorexia nervosa, bulimia nervosa, or compulsive eating)?
☐ Yes, and I have advanced knowledge about them
☐ Yes, but I do not have much information about them
☐ No
10. What type of diet do you follow?
☐ Omnivorous diet (both plant foods and foods of animal origin)
☐ Ovo-lacto-vegetarian diet (eggs, dairy products, and plant foods)
☐ Vegetarian diet (plant foods only)
☐ Meat-based diet (meat and fish are staple foods)
☐ Other:
11. How many main meals do you eat per day?
☐ 1
☐ 2
☐ 3
☐ More than 3 main meals per day
12. How many snacks do you eat between main meals?
☐ I only eat main meals
☐ 1
☐ 2
☐ 3
☐ More than 3 snacks per day
13. What is your weekly level of physical activity?
☐ Sedentary: no physical activity
☐ Light: 1–2 times per week
☐ Moderate: 3–4 times per week
☐ High: 5–7 times per week
14. How satisfied are you with your appearance?
☐ Not at all satisfied
☐ Satisfied
☐ Very satisfied
☐ My appearance is not important to me
15. Have you recently experienced any of the following? *(Select all that apply.)*
☐ Social anxiety
☐ Stress
☐ Depression
☐ Sleep disturbances

☐ Suicidal thoughts
☐ None of the above
☐ Other:
16. Do you usually wake up during the night to eat?
☐ No
☐ Sometimes
☐ Often
17. Which methods have you used to lose weight or prevent weight gain? *(Select all that apply.)*
☐ Self-induced vomiting
☐ Medication
☐ Dietary restriction
☐ Physical exercise
☐ I have not used any such methods
☐ Other:
18. How often do you usually weigh yourself?
☐ Before and after every meal
☐ Once a day
☐ Weekly
☐ Rarely
☐ I do not usually weigh myself
19. How do you choose the foods you eat? *(Select all that apply.)*
☐ I carefully read food labels
☐ I eat the same foods every day because I consider them safe
☐ I select foods according to smell, colour, or texture
☐ I always calculate nutritional values according to the recommended caloric intake
☐ I do not consider this aspect important
☐ Other:
20. Have you ever felt the urge to consume non-food substances (e.g., ice, soil, or paper)?
☐ Yes
☐ No
Section C. Eating, Weight, and Body-Image Statements
Response scale: 0 = Never; 1 = Rarely; 2 = Sometimes; 3 = Often; 4 = Always.
21. The thought that I might weigh too much frightens me.
☐ 0 Never ☐ 1 Rarely ☐ 2 Sometimes ☐ 3 Often ☐ 4 Always
22. I avoid eating when I am hungry because I do not want to gain weight.
☐ 0 Never ☐ 1 Rarely ☐ 2 Sometimes ☐ 3 Often ☐ 4 Always
23. People around me have told me that I should eat more.
☐ 0 Never ☐ 1 Rarely ☐ 2 Sometimes ☐ 3 Often ☐ 4 Always
24. People around me have told me that I should eat less.
☐ 0 Never ☐ 1 Rarely ☐ 2 Sometimes ☐ 3 Often ☐ 4 Always
25. I feel very guilty after eating.
☐ 0 Never ☐ 1 Rarely ☐ 2 Sometimes ☐ 3 Often ☐ 4 Always
26. I sometimes lose control and eat even when I am not hungry.
☐ 0 Never ☐ 1 Rarely ☐ 2 Sometimes ☐ 3 Often ☐ 4 Always
27. I find it difficult to look at myself in the mirror because I do not like what I see.
☐ 0 Never ☐ 1 Rarely ☐ 2 Sometimes ☐ 3 Often ☐ 4 Always
28. I often compare my appearance with what I see on social media (e.g., models or actors).

☐ 0 Never ☐ 1 Rarely ☐ 2 Sometimes ☐ 3 Often ☐ 4 Always

29. I believe that social media negatively influences how I perceive my appearance.

☐ 0 Never ☐ 1 Rarely ☐ 2 Sometimes ☐ 3 Often ☐ 4 Always

Section D. Additional Psychosocial and Eating-Related Statements

30. Please select only the statements that apply to you: *(Select all that apply.)*

☐ I have received negative comments about my appearance, and this has affected my self-confidence.
☐ My parents or family members have insulted me or used words intended to humiliate me.
☐ I receive the support I need from my family.
☐ My schoolmates treated me badly.
☐ I have not had genuine or trustworthy friends.
☐ I cannot achieve my ideal body shape unless I have complete control over my eating.
☐ I tend to continue eating even when I am not hungry.
☐ When I feel sad or stressed, I eat without realising it.
☐ I have previously sought help from a psychotherapist.
